# Accuracy and Reliability of Remote Categorization of Upper Limb Outcome After Stroke

**DOI:** 10.1177/15459683241231272

**Published:** 2024-02-15

**Authors:** Harry T. Jordan, Cathy M. Stinear

**Affiliations:** 1 Clinical Neuroscience Laboratory, Department of Medicine, The University of Auckland, Auckland, New Zealand

**Keywords:** stroke, upper limb, paresis, home-based assessment, remote assessment, outcome measures

## Abstract

**Background:**

There is an increasing need for motor assessments after stroke that can be performed quickly and remotely. The Fast Outcome Categorization of the Upper Limb after Stroke-4 (FOCUS-4) assessment remotely classifies upper limb outcome into 1 of 4 categories after stroke and was developed via retrospective analysis of Action Research Arm Test (ARAT) scores.

**Objective:**

The aim of this study was to prospectively evaluate the accuracy and reliability of FOCUS-4 assessments for categorizing upper limb outcome after stroke when administered remotely during a videocall compared to an in-person ARAT.

**Methods:**

Data were collected from 26 participants at 3 months post-stroke (3M), 27 participants at 6 months post-stroke (6M), and 56 participants at the chronic stage of stroke (>6M). Participants performed an in-person ARAT and a remote FOCUS-4 assessment administered during a videocall, and accuracy was evaluated by comparing the upper limb outcome categories. Participants at the chronic stage of stroke also performed a second remote FOCUS-4 assessment to assess between-day reliability.

**Results:**

Overall accuracy of the remote FOCUS-4 assessment was 88% at 3M and 96% at 6M. Overall accuracy of the first and second remote FOCUS-4 assessments at the chronic stage was 75% and 79%, respectively. Reliability of the FOCUS-4 assessment at the chronic stage was 82%. The remote FOCUS-4 assessment was most accurate and reliable for participants with mild or severe upper limb functional impairment.

**Conclusions:**

The remote FOCUS-4 assessment has potential to classify upper limb functional capacity or to screen possible participants for stroke trials, but external validation is required.

## Introduction

The recovery of upper limb motor function is critical for patients to regain independence after stroke.^1,2^ The Action Research Arm Test (ARAT) is a 19-item in-person assessment of upper limb capacity that is often used in stroke rehabilitation research. The ARAT is the only assessment of upper limb activity limitation recommended by international consensus for stroke rehabilitation trials.^3^

The ARAT score has been used to categorize upper limb functional outcome after stroke in several classification systems.^4-6^ One classification system uses the ARAT to categorize upper limb outcome after stroke into 1 of 4 categories; Excellent, Good, Limited, and Poor. The ARAT cut-off scores for the 4 categories were determined using a hypothesis-free cluster analysis.^5^ People with stroke in these categories report significantly different amounts of paretic upper limb use at 3 months post-stroke, indicating the categories are clinically meaningful.^7^ The same 4 categories have been used by several other research groups to characterize upper limb outcome at 3 months post-stroke.^8,9^

**Figure 1.**
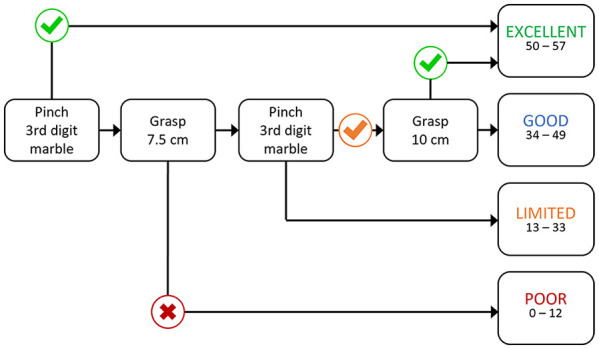
The FOCUS-4 assessment for categorizing upper limb outcome after stroke. Following each task the patient can progress through the assessment via 1 of 2 possible paths. One path has a symbol and the other path does not. The symbol indicates what score the patient needs to achieve to take that path and if the participant does not achieve that score, they will take the path without the symbol. Green ticks indicate a score of 3 for the specified task, orange ticks indicate a score of 2 or 3, and red crosses indicate a score of 0. Participants proceed through the decision tree until an outcome category is reached. The outcome boxes on the right provide ARAT score ranges.

The coronavirus disease-2019 pandemic encouraged the development of assessments that can be performed quickly and remotely.^10^ Several shortened versions of the ARAT have been introduced recently, and all provide an exact score.^11-13^ We previously introduced the Fast Outcome Categorization of the Upper Limb after Stroke-4 (FOCUS-4) assessment for classifying upper limb outcome into Excellent, Good, Limited, and Poor categories (Figure 1).^14^ The FOCUS-4 assessment is unique among shortened versions of the ARAT as it classifies participants into outcome categories corresponding to a range of ARAT scores rather than providing an exact score, and it was developed with the intention to be administered and scored remotely during a videocall. However, the accuracy and reliability of the remote FOCUS-4 assessment for categorizing upper limb outcome after stroke has yet to be investigated.

The current study had 2 aims. The first and primary aim was to evaluate the accuracy of the remote FOCUS-4 assessment for categorizing upper limb outcome after stroke when compared to the ARAT performed in-person. The accuracy of the FOCUS-4 assessment was investigated at 3 and 6 months post-stroke as well as the chronic stage of stroke. The second study aim was to investigate the between-day reliability of the remote FOCUS-4 assessment when used at the chronic stage of stroke. This was done by comparing the upper limb outcome from 2 remote FOCUS-4 assessments. The current study was exploratory and without *a priori* hypotheses.

## Methods

### Participants

Data were collected from 2 prospective studies and data utilized in this study are available from the corresponding author upon reasonable request. Assessments were made at 3 months post-stroke (3M) and 6 months post-stroke (6M) in 1 study as recommended by international consensus^3^ and at the chronic stage of stroke in a separate study (>6M). Some participants took part in both studies and so the data at each time point come from overlapping but not identical samples. Participants assessed at 3M and 6M were consecutively recruited from Auckland City Hospital within 1-week post-stroke between May 2019 and June 2021. These participants were recruited as part of a larger longitudinal observational study investigating the accuracy of Predict Recovery Potential algorithm (PREP2) predictions^5^ made in clinical practice and so there was no planned sample size for participants completing the FOCUS-4 remote assessments. Patients were eligible if they were at least 18 years old, had upper limb weakness from the index stroke, and received a PREP2 prediction for upper limb motor outcome as part of routine clinical care. This study was approved by the regional ethics committee. The second prospective study included a convenience sample of people with chronic stroke to compare in-person and remote upper limb outcome measures. There was no prior work on which to base a sample size for the study. Potential participants were identified using several stroke research databases and people were eligible if they were aged 18 years or older, were at least 6 months since their most recent stroke, self-reported they were still experiencing upper limb weakness resulting from stroke, and could participate in a videocall with help from a friend or family member. Five participants who reported no upper limb weakness but who met the other inclusion criteria were also included. This chronic stroke study was approved by the institutional ethics committee. Both studies excluded people if they lived outside the Auckland region, if their stroke occurred in the cerebellum, or if they had pre-existing conditions or cognitive impairments precluding informed consent and compliance with study assessments. All participants in both studies provided written informed consent.

### Procedure

Participants recruited within 1-week post-stroke performed the ARAT in-person and the FOCUS-4 assessment remotely at 3 and/or 6 months post-stroke. The in-person and remote assessments were planned to be separated by at least 48 hours. The order of the 2 assessments was randomized but was not always possible to adhere to for practical reasons. Participants with chronic stroke performed a remote FOCUS-4 assessment followed by an in-person ARAT at least 48 hours later. A second remote FOCUS-4 assessment was performed at least 48 hours after the in-person ARAT. Thus, participants with chronic stroke performed the ARAT in-person once and the FOCUS-4 assessment remotely twice. The in-person ARAT score was used as a comparison for both remote FOCUS-4 assessments. Participants in both studies also completed the Fugl-Meyer Upper Extremity assessment with their paretic upper limb during the in-person assessment to characterize upper limb impairment.^15^

In-person assessments were completed at a location of the participant’s choosing while participants performed the remote assessments from a location of their choosing while the assessor was in a different location. Remote assessments were performed using a videocalling application of the participant’s choosing and the videocalls were not recorded because the assessor scored the tasks in real-time. Participants were seated upright at a table when performing the ARAT and FOCUS-4 assessments. Trained assessors administered all assessments. A single assessor administered all the in-person and remote assessments except for 5 and 11 in-person assessments for the 3M and 6M cohorts, respectively.

Pre-stroke handedness was self-reported for participants recruited within 1-week post-stroke. Pre-stroke handedness for participants with chronic stroke was determined using the short-form of the Edinburgh Handedness Inventory.^16^ Right- and left-handedness was determined by scores greater and less than 0, respectively.

### Action Research Arm Test

The ARAT consists of 19 tasks grouped into grasp, grip, pinch, and gross subscales.^17^ Each task is scored ordinally from 0 to 3 and higher scores indicate better upper limb functional capacity. A score of 3 indicates the task is completed normally. A score of 2 indicates the task is completed but the participant uses compensatory movements or takes abnormally long. A score of 1 indicates movement is partially performed but the task is not completed. A score of 0 indicates the participant cannot perform any part of the task. The ARAT was scored according to a standardized protocol and published time limits from older adults without stroke were used to determine whether task completion was abnormally long.^18,19^ The upper limb outcome for participants with ARAT scores of 0 to 12 was categorized as Poor, scores of 13 to 33 as Limited, scores of 34 to 49 as Good, and scores of 50 to 57 as Excellent as used in previous research.^5,8,9^

### FOCUS-4 Assessment

The FOCUS-4 assessment is shown in Figure 1 and a detailed description of its development has been reported previously.^14^ Briefly, the FOCUS-4 assessment consists of 3 tasks derived from the ARAT. The same scoring rules for the ARAT are used for the FOCUS-4 assessment so each task is scored ordinally from 0-3. Assessors score each task during the videocall. Tasks are ordered in a decision tree and participants progress through it starting with the “Pinch third digit marble” task until an upper limb outcome is reached. Following each task the participant can progress through the assessment via 1 of 2 possible paths. One path has a symbol and the other path does not. The symbol indicates what score the participant needs to achieve to take that path and if the participant does not achieve that score they will take the path without the symbol. Green ticks indicate a score of 3 for the specified task, orange ticks indicate a score of 2 or 3, and red crosses indicate a score of 0. For example, if the participant scores a 3 on the first task “Pinch third digit marble” they follow the path with the green tick symbol. This means the participant is categorized as Excellent which corresponds to an ARAT score range of 50 to 57 and the FOCUS-4 assessment is complete. If the participant scores 0, 1, or 2 on the first task “Pinch third digit marble” they follow the path with no symbol and then perform the “Grasp 7.5 cm cube” task. The participant continues along the decision tree until an upper limb outcome is reached. The outcome boxes on the right provide ARAT score ranges for each outcome category.

The order of the remote tasks did not follow the order of the FOCUS-4 decision tree for the current study. Rather, participants performed the 10 and 7.5 cm grasp tasks in that order followed by all the pinch tasks. This was done because separate FOCUS-3 and FOCUS-5 assessments were also being evaluated, but the results for these assessments are not presented here.^14^ Participants’ upper limb outcome was categorized as Excellent, Good, Limited, or Poor by applying the FOCUS-4 assessment decision tree to their individual task scores.

The items required to perform the FOCUS-4 assessment were delivered to participants, and these items are displayed in Figure I in the Supplemental Materials. Items for the FOCUS-4 assessment replicated ARAT item specifications.^18^ Cardboard boxes filled with sand were used to replicate the dimensions and mass of the 10 and 7.5 cm wooden blocks in the ARAT. A marble and 2 jar lids were used for the pinch task while a postage box with a height of 35 cm was used to replicate the table-shelf in the ARAT.

A friend or family member was present with the participant during the remote FOCUS-4 assessment to hold the videocall device so the participant could perform the tasks. Participants were told how to set up the items for each task and were given instructions for completing each task during the videocall. The remote FOCUS-4 assessment used the same task-specific object positioning and instructions as the ARAT.^18^ A comparison of the set up for the “grasp 7.5 cm cube” task performed as part of the ARAT and FOCUS-4 assessments is shown in Figure II in the Supplemental Materials.

### Data Analysis

Upper limb outcomes using the ARAT and FOCUS-4 assessment were only determined after both the in-person and remote assessments had been completed to minimize potential assessor bias. Overall accuracy of the FOCUS-4 assessment at 3M, 6M, and the chronic stage of stroke was calculated as the number of participants with the same outcome from the ARAT and FOCUS-4 assessments divided by the total number of participants. The positive predictive values and negative predictive values were calculated for each outcome category and are provided in Tables I to IV in the Supplemental Materials. The reliability of the FOCUS-4 assessment at the chronic stage of stroke was calculated as the number of participants with the same outcome category from both FOCUS-4 assessments divided by the total number of participants. All analyses were exploratory and so no pre-specified thresholds for interpretation were used.

## Results

There were 26 participants assessed at 3M, 27 participants at 6M, and 56 participants at the chronic stage of stroke. Twenty participants were assessed at both 3M and 6M, 1 participant was assessed at 6M and the chronic stage, and 7 participants were assessed at all 3 timepoints. Participants who were part of both the 6M and chronic stroke cohorts had at least a 6-week gap between evaluations. A single assessor administered all assessments except for 5 and 11 in-person assessments at 3M and 6M, respectively. For practical reasons, 69% of participants at the 3M and 6M timepoints performed the ARAT first. Participant characteristics at each timepoint are provided in Table 1.

**Table 1.** Participant Characteristics in Each Cohort.

	3M post-stroke (n = 26)	6M post-stroke (n = 27)	Chronic stroke (n = 56)
Age mean ± SD (years, range)	73 ± 16 (41 - 99)	68 ± 14 (41 - 91)	66 ± 14 (29 - 89)
Sex (M, F)	17, 9	18, 9	32, 24
Handedness (R, %)	22 (85%)	24 (89%)	52 (93%)
Dominant upper limb affected (%)	14 (54%)	9 (33%)	25 (45%)
Months since stroke mean ± SD (range)	2.9 ± 0.1 (2.7 - 3.4)	6.2 ± 0.3 (5.9 - 6.9)	37.5 ± 51.2 (7.0 - 235.0)
Days between assessments mean ± SD (range)	5 ± 4 (0 - 14)	4 ± 5 (0 - 24)	19 ± 47 (4 - 324)
ARAT score mean ± SD (range)	37 ± 23 (0 - 57)	33 ± 26 (0 - 57)	36 ± 21 (0 - 57)
FM-UE score mean ± SD (range)	45 ± 22 (8 - 66)	42 ± 24 (8 - 66)	46 ± 19 (9 - 66)

Abbreviations: 3M, 3 months post-stroke; 6M, 6 months post-stroke; ARAT, Action Research Arm Test; FM-UE, Fugl-Meyer upper extremity; SD, standard deviation.

### FOCUS-4 Assessment Accuracy

The overall accuracy of the remote FOCUS-4 assessment at 3M was 88% (Table 2). Each of the 3 participants misclassified by the remote FOCUS-4 assessment were misclassified into an outcome category 1 better than their ARAT outcome. Accuracy was 100% for Limited and Poor outcome categories, however there was only 1 participant in the Limited category (Table I in Supplemental Materials).

**Table 2.** 3M Accuracy of the Remote FOCUS-4 Assessment for Determining Upper Limb Outcome Compared to the in-person ARAT.

	Remote FOCUS-4 assessment			
	Excellent	Good	Limited	Poor
In-person ARAT				
Excellent	13	0	0	0
Good	2	2	0	0
Limited	0	1	1	0
Poor	0	0	0	7
PPV (%)	86.7	66.7	100	100

Abbreviations: 3M, 3 months post-stroke; ARAT, Action Research Arm Test; FOCUS-4, Fast Outcome Categorization of the Upper Limb After Stroke-4; PPV, positive predictive value.

The overall accuracy of the FOCUS-4 assessment at 6M was 96% as only 1 participant was miscategorized (Table 3). No participants at 6M were categorized as Limited using the ARAT or FOCUS-4 assessment (Table II in Supplemental Materials).

**Table 3.** 6M Accuracy of the Remote FOCUS-4 Assessment for Determining Upper Limb Outcome Compared to the In-Person ARAT.

	Remote FOCUS-4 assessment			
	Excellent	Good	Limited	Poor
In-person ARAT				
Excellent	13	0	0	0
Good	1	3	0	0
Limited	0	0	0	0
Poor	0	0	0	10
PPV (%)	92.9	100	—	100

Abbreviations: 6M, 6 months post-stroke; ARAT, Action Research Arm Test; FOCUS-4, Fast Outcome Categorization of the Upper Limb After Stroke-4; PPV, positive predictive value.

For participants with chronic stroke the overall accuracy of the first and second FOCUS-4 assessments were 75% and 79%, respectively (Table 4). Two people were misclassified by both FOCUS-4 assessments as Excellent when they were categorized as Good by the ARAT. Four people categorized as Limited by the ARAT were miscategorized by both FOCUS-4 assessments as either Good or Poor (Tables III and IV in Supplemental Materials).

**Table 4.** Chronic Stage of Stroke. Accuracy of the first and second remote FOCUS-4 assessments for determining upper limb outcome compared to the in-person ARAT.

		First remote FOCUS-4 assessment				Second remote FOCUS-4 assessment			
		Excellent	Good	Limited	Poor	Excellent	Good	Limited	Poor
In-person ARAT									
Excellent (n = 22)		21	1	0	0	22	0	0	0
Good (n = 12)		3	6	3	0	4	8	0	0
Limited (n = 8)		0	2	3	3	0	4	2	2
Poor (n = 14)		0	0	2	12	0	0	2	12
PPV (%)		87.5	66.7	37.5	80.0	84.6	66.7	50.0	85.7

Abbreviations: n, number of participants in category using in-person ARAT categorization; ARAT, Action Research Arm Test; FOCUS-4, Fast Outcome Categorization of the Upper Limb after Stroke-4; PPV, positive predictive value.

### FOCUS-4 Assessment Reliability

The median number of days between remote FOCUS-4 assessments for participants with chronic stroke was 14 days with a range of 7 to 647 days. Both remote assessments were performed within 28 days for 82% of participants.

Repeated FOCUS-4 assessments classified 82% of participants with chronic stroke into the same category (Table 5). Reliability was highest for the Excellent and Poor categories. Nine of the 10 participants who were classified differently by the 2 FOCUS-4 assessments were classified into a better outcome category during the second FOCUS-4 assessment compared to the first. Eight participants were classified into the same category using both FOCUS-4 assessments but into a different category using the ARAT.

**Table 5.** Chronic Stage of Stroke. Comparison of upper limb outcomes determined using the first and second remote FOCUS-4 assessments.

	Second remote FOCUS-4 assessment			
	Excellent	Good	Limited	Poor
First remote FOCUS-4 assessment				
Excellent	23	1	0	0
Good	3	6	0	0
Limited	0	5	3	0
Poor	0	0	1	14

Abbreviation: FOCUS-4, Fast Outcome Categorization of the Upper Limb after Stroke-4.

## Discussion

This study investigated the accuracy and between-day reliability of the remote FOCUS-4 assessment for categorizing upper limb motor outcome after stroke when administered during a videocall. Overall, the FOCUS-4 assessment was 88% and 96% accurate at categorizing upper limb outcome at 3 and 6 months after stroke, respectively. Accuracy at the chronic stage of stroke was 75% and 79% for the first and second FOCUS-4 assessments, respectively. The FOCUS-4 assessment was most accurate and reliable at categorizing participants with mild or severe upper limb functional impairment but was less accurate and reliable for participants with moderate functional impairment. These findings provide evidence the remote FOCUS-4 assessment may be useful for remotely categorizing upper limb outcomes of people with mild or severe upper limb functional impairment at the sub-acute and chronic stages of stroke. In the future the FOCUS-4 assessment could be used to categorize upper limb outcomes remotely in research as well as screen potential participants for upper limb rehabilitation trials.

### Accuracy of Remote FOCUS-4 Assessment

The remote FOCUS-4 assessment demonstrated high overall accuracy at 3 and 6 months post-stroke but was less accurate at the chronic stage of stroke. It is possible that the lower accuracy at the chronic stage of stroke was related to the degree of functional impairment in each cohort. The proportion of participants categorized as Good or Limited using the ARAT was higher for the chronic cohort (36%) than for the 3 months (23%) or 6-month cohorts (15%), and the FOCUS-4 assessment was least accurate at classifying people into these 2 categories. The relatively low proportion of participations in the Good or Limited categories at 6 months post-stroke could also contribute to the higher overall accuracy at that time point. This could also contribute to the higher overall accuracy at 6 months compared to 3 months post-stroke. Larger sample sizes at all 3 timepoints will be needed to determine whether the lower accuracy of the FOCUS-4 assessment at the chronic stage was at least partially due to the distribution of upper limb severity in the sample populations.

At each timepoint the FOCUS-4 assessment was most accurate for participants at the outer ranges of upper limb functional capacity with Excellent or Poor outcomes. The same finding was reported during the development of the FOCUS-4 assessment using retrospective data.^14^ Several participants at each time point who were categorized as Excellent with the FOCUS-4 assessment were categorized as Good with the ARAT. The misclassified participants were likely due to chance as they had no obvious shared characteristics and their total ARAT scores were spread throughout the range for the Good category.

At the other end of the impairment spectrum, the remote FOCUS-4 assessment was 100% accurate classifying participants into the Poor category at 3 and 6 months post-stroke and 86% accurate at the chronic stage of stroke. This high accuracy is likely because participants in the Poor category have little to no voluntary movement in their upper limb and so cannot even partially complete tasks during either the ARAT or FOCUS-4 assessment. The remote aspect of the FOCUS-4 assessment, in addition to other slight differences in the items used, were therefore unlikely to have affected these participants’ task performance. Two participants with chronic stroke who were classified as Poor using both remote FOCUS-4 assessments were categorized as Limited using the ARAT. Both these participants were categorized as Poor using the FOCUS-4 assessment because they scored zero on the “grasp 7.5 cm cube.” However, they also scored zero on the same task using the ARAT which indicates the misclassification was not due to a problem with performing or scoring the FOCUS-4 assessment remotely. Rather, both participants scored well enough on easier ARAT tasks, such as grasping 2.5 and 5 cm cubes, to exceed the ARAT score range for the Poor category. Thus, the misclassification for these participants is likely because they did not have enough finger extension to grasp the 7.5 cm cube in the FOCUS-4 assessment but had enough movement to at least partially complete easier ARAT tasks. Overall, the current findings indicate that upper limb outcome can be reliably determined using the FOCUS-4 assessment for participants with mild or severe upper limb functional impairment.

The remote FOCUS-4 assessment was less accurate at categorizing participants in the Good and Limited categories than in the Excellent and Poor categories. The retrospective analysis for developing the FOCUS-4 assessment also found the Good category was the least accurate category to classify participants into.^14^ The small number of participants in the Limited category relative to the other categories makes it difficult to draw conclusions as only 10 participants were categorized as Limited using the ARAT across the 3 timepoints. The low number of participants in the Limited category is not surprising as 2 other studies combined found only 20 out of 140 participants with stroke had an ARAT score in the Limited category range at 3 months after stroke.^8,9^ Similarly, a retrospective analysis of 3,738 ARAT scores found only 22% had a score from 11 to 42.^13^ The low accuracy for the Limited category may be related to the fact only 29 of the 333 of ARAT scores used to develop the FOCUS-4 assessment were in the Limited category range.^14^ A larger sample of ARAT scores within the Limited range may be needed to find a FOCUS-4 task that more accurately categorizes these participants. The current findings indicate the remote FOCUS-4 assessment may not be accurate for determining upper limb outcome for participants with moderate upper limb functional impairment, and this requires evaluation in a larger sample.

### Reliability of Remote FOCUS-4 Assessment

Overall, 82% of participants with chronic stroke were classified into the same category by both remote FOCUS-4 assessments. Nine of the 10 participants who were classified differently by the 2 FOCUS-4 assessments were classified into a better outcome category during the second assessment. It is unlikely this resulted from underlying improvement in upper limb performance as the FOCUS-4 assessments were separated by fewer than 3 weeks for 7 of these 9 participants. A more likely possibility is that participants were more familiar with the FOCUS-4 tasks and videocall setup on their second assessment. Thirty-seven of the 39 participants with chronic stroke who were classified as Excellent or Poor with the first FOCUS-4 assessment were classified into the same outcome category with the second assessment. The between-day reliability results agree with the accuracy results that participants with mild and severe upper limb functional impairment are the easiest to categorize using the FOCUS-4 assessment while participants with moderate upper limb functional capacity are the most difficult to categorize.

### Applications for the Remote FOCUS-4 Assessment

The remote FOCUS-4 assessment has several potential future applications. There has been an increasing desire for stroke assessments that can be performed quickly and remotely.^10^ There is also growing interest in telerehabilitation and trials delivering stroke interventions remotely,^20,21^ and telerehabilitation is expected to become commonly used in the future.^22^ Although there is evidence showing telerehabilitation is not inferior to in-person rehabilitation after stroke, the quality of evidence is low-to-moderate and further studies of telerehabilitation and remote interventions are needed.^23,24^ A simple behavioral assessment for remotely evaluating a participant’s upper limb functional outcome such as the FOCUS-4 assessment could increase the viability of telerehabilitation research and services. The option of remote assessment could allow people to participate in telerehabilitation services and research trials without needing any in-person contact. This could increase people’s willingness to participate and stay in research, and providing alternative methods of data collection improves retention rates in longitudinal clinical trials.^25^

The option of remote assessment could also increase research opportunities for people living rurally who often decline to participate in, or are excluded from, clinical trials due to the need for in-person assessments.^26-28^ Stroke telerehabilitation studies have successfully been used with people living remotely.^29^ It is especially important to include more people living rurally in research because they have a higher stroke incidence and mortality, have worse access to hyperacute stroke interventions, and experience worse overall outcomes post-stroke compared to people living in urban areas.^30-32^

The FOCUS-4 assessment could also aid study recruitment. Chronic stroke studies commonly have ARAT score cutoffs as inclusion criteria,^33-37^ and being able to accurately categorize people at the outer ranges of functional capacity using a short 5-minute videocall could help screen potential participants. For example, a study by Rodgers et al^33^ using robot assisted training for the upper limb after stroke only included people with an ARAT score between 0 and 39. A small box of items, similar to the one shown in Figure I in the Supplemental Materials, could be posted to potential participants. Performing a FOCUS-4 assessment remotely with potential participants would then allow researchers to exclude anyone categorized as Excellent using the FOCUS-4 assessment remotely because this category corresponds to an ARAT score between 50 and 57. Participants who subsequently enroll in the study could then return the box of items when they meet the researchers in-person. This method would save travel and assessment time for both the researchers and potential participants. Potential applications of the FOCUS-4 assessment could be evaluated in future studies.

### Limitations, Strengths, and Future Directions

This study had several limitations, one being the small sample sizes at 3 and 6 months post-stroke. Additionally, the distribution of participants was skewed toward the outer ranges of functional capacity. A third limitation is that the FOCUS-4 assessment tasks were not performed in the intended order using the decision tree structure, and the effect of task order on remote FOCUS-4 accuracy could be explored in the future. A further limitation is the potential for bias because most participants were assessed by the same person for the in-person and remote assessments.

A strength of the study is that the remote FOCUS-4 assessment demonstrated high overall accuracy at 3separate timepoints after stroke. This includes high accuracy at 3 months after stroke, and all stroke intervention trials are recommended to include an outcome assessment at this timepoint.^3^ Another strength is that participants with chronic stroke had at least 48 hours between assessments to reduce any practice effects, although this was not always feasible with participants at 3 and 6 months post-stroke. Lastly, interrater variance was minimized by having the same assessor perform most of the remote and in-person assessments.

The results from the current study reveal several future directions. The accuracy of the remote FOCUS-4 assessment for determining upper limb outcome needs to be evaluated with a larger number of participants and particularly those with moderate upper limb functional impairment. Interrater reliability of the FOCUS-4 assessment needs to be determined in addition to between-day reliability at 2 and 6 months after stroke. Moreover, external validation of the FOCUS-4 assessment is needed to assess its reproducibility and generalizability in other environments.^38^ The feasibility of using remote upper limb assessments with people with stroke could be investigated to identify barriers and facilitators that are expected to vary across cultural and socioeconomic environments, with a view to making remote assessments more accessible for participants. The same questions regarding feasibility are also needed for telerehabilitation studies which have largely focused on clinical effectiveness.^39^ Finally, it remains to be determined whether performing tasks in the intended order using the decision tree structure affects accuracy of the remote FOCUS-4 assessment.

## Conclusion

This study provided evidence that the remote FOCUS-4 assessment can accurately categorize upper limb functional outcome for people after stroke with mild or severe upper limb functional impairment. Future research is needed to confirm the current findings in a larger group of participants, particularly those with moderate upper limb functional capacity. In the future remote FOCUS-4 assessment could be used to categorize upper limb functional outcome after stroke or to screen potential participants for inclusion in stroke studies.

## Supplemental Material

sj-docx-1-nnr-10.1177_15459683241231272 – Supplemental material for Accuracy and Reliability of Remote Categorization of Upper Limb Outcome After Stroke Supplemental material, sj-docx-1-nnr-10.1177_15459683241231272 for Accuracy and Reliability of Remote Categorization of Upper Limb Outcome After Stroke by Harry T. Jordan and Cathy M. Stinear in Neurorehabilitation and Neural Repair
