# Supplementary material for: Accuracy and Reliability of Remote Categorization of Upper Limb Outcome After Stroke
Source: Neurorehabil Neural Repair. 2024 Feb 15;38(3):167–75. doi: 10.1177/15459683241231272 (PMC10943605; doi:10.1177/15459683241231272)
Supplement: sj-docx-1-nnr-10.1177_15459683241231272 – Supplemental material for Accuracy and Reliability of Remote Categorization of Upper Limb Outcome After Stroke [file sj-docx-1-nnr-10.1177_15459683241231272.docx]

**SUPPLEMENTAL MATERIALS**

Accuracy and Reliability of Remote Categorization of Upper Limb Outcome after Stroke

Supplemental Tables

Table I. 3M after stroke. Individual outcome category accuracies for the remote FOCUS-4 assessment.

|  | **Accuracy (95% CI)** | **PPV (95% CI)** | **NPV (95% CI)** |
| --- | --- | --- | --- |
| Excellent (n = 13) | 92.3%(74.9 – 99.1%) | 86.7%(64.5 – 95.9%) | 100.0%(100.0 – 100.0%) |
| Good (n = 4) | 88.5%(69.9 – 97.6%) | 66.7%(18.9 – 94.5%) | 91.3%(79.7 – 96.6%) |
| Limited (n = 2) | 88.5%(69.9 – 97.6%) | 33.3%(6.8 – 77.3%) | 95.7%(84.6 – 98.9%) |
| Poor (n = 7) | 100.0%(86.8 – 100.0%) | 100.0%(100.0 – 100.0%) | 100.0%(100.0 – 100.0%) |

n = number of participants in category using in-person Action Research Arm Test categorization. CI, confidence interval; FOCUS-4, Fast Outcome Categorization of the Upper Limb after Stroke-4; NPV, negative predictive value; PPV, positive predictive value.

Table II. 6M after stroke. Individual outcome category accuracies for the remote FOCUS-4 assessment.

|  | **Accuracy (95% CI)** | **PPV (95% CI)** | **NPV (95% CI)** |
| --- | --- | --- | --- |
| Excellent (n = 13) | 96.3% (81.0 – 99.9%) | 92.9% (66.3 – 98.9%) | 100.0% (100.0 – 100.0%) |
| Good (n = 4) | 96.3% (81.0 – 99.9%) | 100.0% (100.0 – 100.0%) | 95.8% (80.8 – 99.2%) |
| Limited (n = 0) | -- | -- | -- |
| Poor (n = 10) | 100.0% (87.2 – 100.0%) | 100.0% (100.0 – 100.0%) | 100.0% (100.0 – 100.0%) |

n = number of participants in category using in-person Action Research Arm Test categorization. CI, confidence interval; FOCUS-4, Fast Outcome Categorization of the Upper Limb after Stroke-4; NPV, negative predictive value; PPV, positive predictive value.

Table III. Chronic stage after stroke. Individual outcome category accuracies for the first remote FOCUS-4 assessment.

|  | **Accuracy (95% CI)** | **PPV (95% CI)** | **NPV (95% CI)** |
| --- | --- | --- | --- |
| Excellent (n = 22) | 92.9% (82.7 – 98.0%) | 87.5% (70.3 – 95.4%) | 96.9% (82.0 – 99.5%) |
| Good (n = 12) | 83.9% (71.7 – 92.4%) | 66.7% (36.9 – 87.3%) | 87.2% (79.4 – 92.4%) |
| Limited (n = 8) | 82.1% (69.6 – 91.1%) | 37.5% (15.1 – 67.0%) | 89.6% (83.3 – 93.7%) |
| Poor (n = 14) | 91.1% (80.4 – 97.0%) | 80.0% (56.8 – 92.4%) | 95.1% (84.4 – 98.6%) |

n = number of participants in category using in-person Action Research Arm Test categorization. CI, confidence interval; FOCUS-4, Fast Outcome Categorization of the Upper Limb after Stroke-4; NPV, negative predictive value; PPV, positive predictive value.

Table IV. Chronic stage after stroke. Individual outcome category accuracies for the second remote FOCUS-4 assessment.

|  | **Accuracy (95% CI)** | **PPV (95% CI)** | **NPV (95% CI)** |
| --- | --- | --- | --- |
| Excellent (n = 22) | 92.9% (82.7 – 98.0%) | 84.6% (68.7 – 93.3%) | 100.0% (88.4 – 100.0%) |
| Good (n = 12) | 85.7% (73.8 – 93.6%) | 66.7% (42.0 – 84.7%) | 90.9% (81.7 – 95.7%) |
| Limited (n = 8) | 85.7% (73.8 – 93.6%) | 50.0% (14.1 – 86.0%) | 88.5% (83.7 – 92.0%) |
| Poor (n = 14) | 92.9% (82.7 – 98.0%) | 85.7% (60.4 – 95.9%) | 95.2% (84.7 – 98.6%) |

n = number of participants in category using in-person Action Research Arm Test categorization. CI, confidence interval; FOCUS-4, Fast Outcome Categorization of the Upper Limb after Stroke-4; NPV, negative predictive value; PPV, positive predictive value.

Supplemental Figures


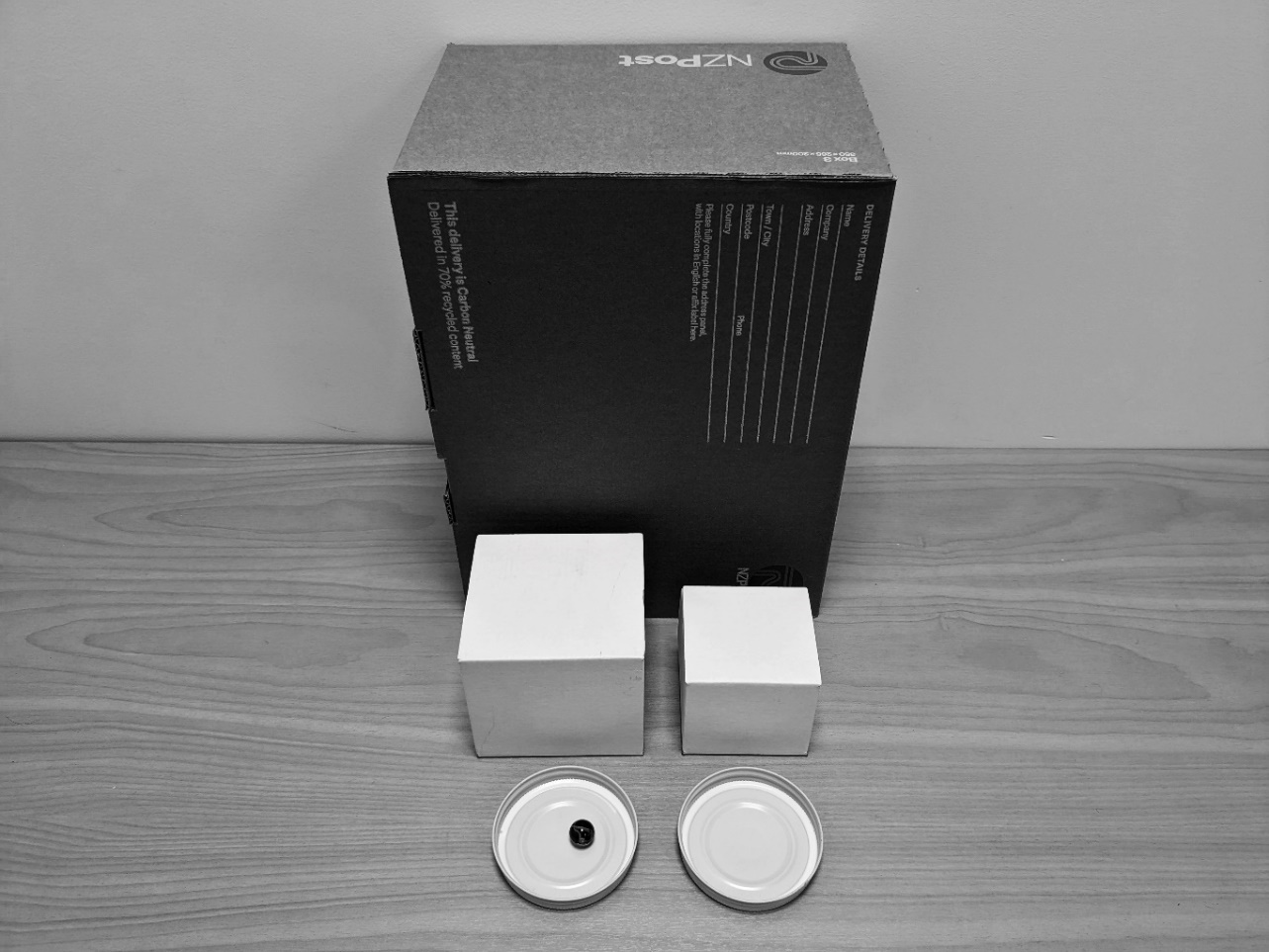


Figure I. Items provided to participants to perform the FOCUS-4 assessment.


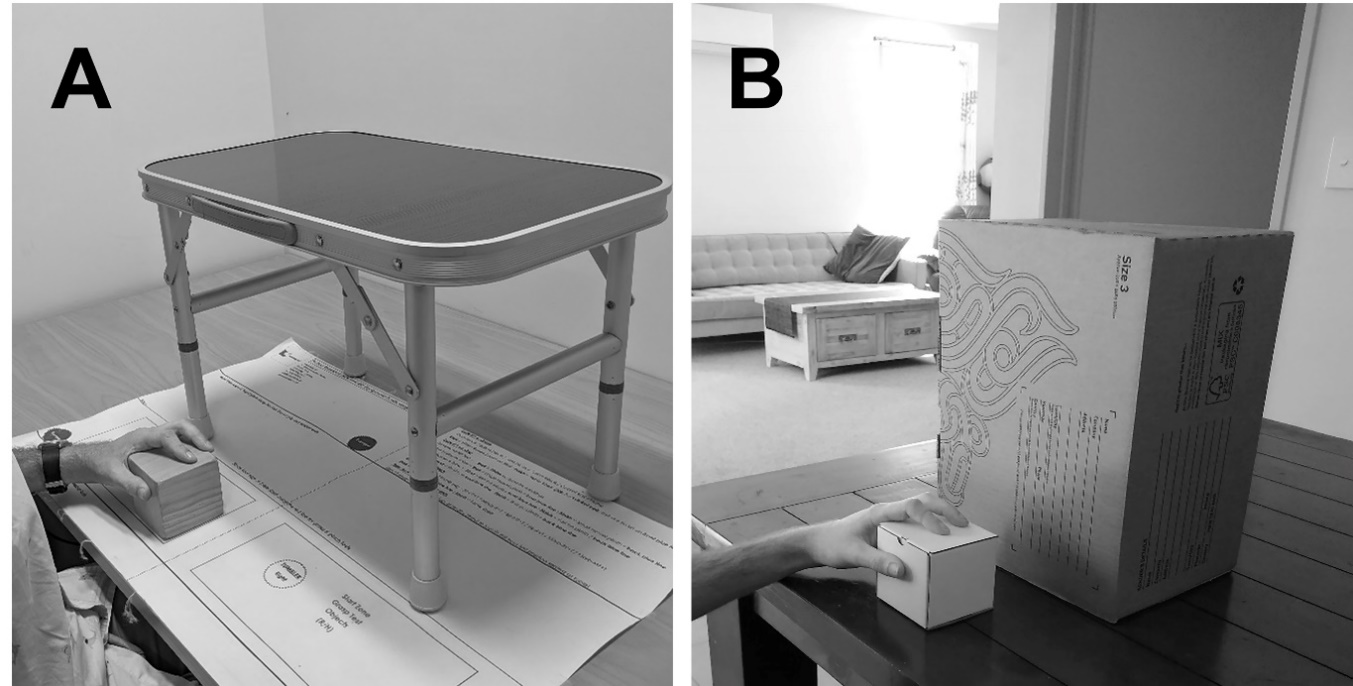
Figure II. Comparison of set-up and items used for the “grasp 7.5 cm cube” task in the in-person ARAT (A) and remote FOCUS-4 assessment (B). Participants must grip the cube with their paretic hand, lift it on top of the table-shelf or postage box, then place their hand back on the table.
